# Erenumab versus topiramate: post hoc efficacy analysis from the HER-MES study

**DOI:** 10.1186/s10194-022-01511-y

**Published:** 2022-11-15

**Authors:** Marc Ehrlich, Christian Hentschke, Christian Sieder, Monika Maier-Peuschel, Uwe Reuter

**Affiliations:** 1 Novartis Pharma GmbH, Nuremberg, Germany; 2 Department of Neurology, Charité Universitätsmedizin Berlin, Charitéplatz 1, 10117 Berlin, Germany; 3 Universitätsmedizin Greifswald, Greifswald, Germany

**Keywords:** Erenumab, Topiramate, Calcitonin gene-related peptide, Preventive, Migraine, Efficacy, Standard of care

## Abstract

**Objective:**

HER-MES was the first head-to-head, phase 4 trial to assess the tolerability and effectiveness of erenumab against standard of care treatment (topiramate). This post hoc analysis compared the efficacy of erenumab with topiramate in patients who completed the trial on study medication.

**Methods:**

Post hoc sensitivity analysis was performed using the full analysis set. Outcomes assessed included the proportion of patients with a ≥50% reduction in monthly migraine days (MMD) from baseline (50% responder rate), over the last 3 months (months 4, 5, and 6) of the double-blind treatment phase (DBTP), the 50% responder rate during the first month of the DBTP, and change from baseline in MMD during the DBTP. Multiple imputation was done for efficacy values of patients who discontinued study treatment.

**Results:**

Patients (*N* = 777) were randomly assigned (1:1) to either 70 or 140 mg/month erenumab (*N* = 389) or 50–100 mg/day topiramate (*N* = 388). Of these, 334 patients (85.9%) receiving erenumab, and 231 patients (59.5%) receiving topiramate completed the DBTP on study medication. Patients on study medication until the end of the DBTP received a mean dose of 119 mg/month for erenumab and 92 mg/day for topiramate. At month 1, a significantly greater proportion of patients receiving erenumab (39.2%) reported ≥50% reduction in MMD from baseline compared with those receiving topiramate (24.0%; *p* < 0.001). In the last 3 months, a significantly larger proportion of patients receiving erenumab (60.3%) achieved ≥50% reduction in MMD from baseline compared with those receiving topiramate (43.3%; *p* < 0.001). Patients receiving erenumab demonstrated significantly greater reductions in MMD during the last 3 months from baseline versus those receiving topiramate (− 6.13 vs − 4.90; 95% CI: − 1.87 to − 0.61; *p* < 0.001).

**Conclusions:**

This post hoc analysis demonstrated significantly superior efficacy of erenumab versus topiramate in achieving a ≥50% reduction in MMD with an early onset of efficacy.

**Trial registration:**

ClinicalTrials.gov NCT03828539.

## Introduction

Globally, migraine is the most burdensome of all neurological diseases and is a leading cause of years lost due to disability [1, 2]. Migraine-induced disability and its impact on patient life is under-recognized and poorly understood, resulting in suboptimal treatment [3–5].

Although current prophylactic medications can reduce headache frequency, duration, and severity, most have been repurposed from other disease states and have not been designed to treat the underlying pathophysiology of migraine [6, 7].

Adherence and persistence to prophylactic therapy are poor, with many patients discontinuing due to safety, tolerability and/or efficacy issues [8–11]. Therefore, patients and physicians are hesitant to try prophylaxis due to the belief that unwanted side effects can occur or based on negative prior experience [12, 13].

Several drugs including beta-blockers, sodium channel modulators (topiramate and valproate) and tricyclic anti-depressants are currently being used for the preventive treatment of migraine [14, 15]. Among the oral prophylactic drugs, topiramate has a proven efficacy and safety profile based on pivotal clinical trials [16–18]. According to international guidelines, topiramate is recommended as a first-line medication for the preventive treatment of migraine [14, 15].

Monoclonal antibodies against calcitonin gene-related peptide (CGRP), or its receptor, offer migraine specific preventive treatment for episodic and chronic migraine (EM/CM). Treatment with erenumab, a monoclonal antibody against the CGRP receptor, was demonstrated to be safe and effective in clinical trials in patients with EM and CM and with previous preventive treatment failures [19–26].

The HER-MES (Head-to-head study of Erenumab against topiRamate – Migraine study to assess tolerability and efficacy in a patiEnt-centered Setting) study (ClinicalTrials.gov Identifier: NCT03828539) reached beyond the gold-standard of efficacy analysis in double-blind placebo-controlled trials by conveying the real-world situation into a randomized, controlled, double-blind, double-dummy, head-to-head trial that compared the effectiveness of erenumab versus topiramate [27].

Tolerability of the study medication was the primary endpoint in the HER-MES study, and efficacy endpoints were analyzed in composite populations with patients on therapy and patients who had stopped medication but continued daily reporting. The results showed a superior effectiveness of erenumab over topiramate by means of better tolerability and higher efficacy [27].

However, when it comes to deciding upon a specific treatment, physicians require data about the actual treatment efficacy in patients who continue on active medication to compare therapies – an unmet need for CGRP pathway inhibitors to date. To address this need, here we describe the post hoc analysis of treatment efficacy of erenumab and topiramate in patients who tolerated the medication and were able to complete the study treatment.

This post hoc efficacy analysis assessed the 50% responder rate and the reduction in monthly migraine days (MMD) based on the results of patients who completed the HER-MES study on the study medications [27].

## Methods

### Study design

HER-MES (NCT03828539) was a 24-week, randomized, double-blind, double-dummy, active-controlled, parallel-group, phase 4 trial conducted in Germany that compared the tolerability and effectiveness of erenumab (70 mg or 140 mg QM) with topiramate (titrated to 50-100 mg/day). The primary endpoint, the proportion of patients who discontinued erenumab or topiramate due to an adverse event (AE) during the double-blind treatment phase (DBTP), has been reported in an earlier publication [27]. Details of the study design, randomization, and primary analysis have been reported previously [27].

### Patients

The eligibility criteria for HER-MES have been previously described [27]. Briefly, HER-MES enrolled adult patients (18–65 years) with a history of EM or CM, as defined by the International Classification of Headache Disorders, 3rd edition [28], for at least 12 months prior to screening.

Patients were eligible if they were either naive to prophylactic migraine treatment or had failed or had not been suitable for up to three of the following treatments: metoprolol/propranolol, amitriptyline, or flunarizine. Eligibility for randomization was assessed based on migraine frequency and electronic diary (eDiary) compliance (≥80% eDiary compliance) during the baseline phase. Patients previously treated with topiramate, any drug targeting the CGRP pathway, valproate, or onabotulinumtoxin A were excluded.

Information about the duration and severity of headache days – migraine and non-migraine – as well as use of rescue medication was recorded in an eDiary by the patients [27].

### Outcomes

The post hoc sensitivity analysis was performed on the full analysis set population (FAS) for pre-specified outcomes: i) the proportion of patients with a ≥ 50% reduction in MMD from baseline (50% responder rate), over the last 3 months (months 4,5 and 6) of the DBTP, ii) the 50% responder rate during the first month of the DBTP and iii) the change from baseline in MMD during the DBTP. Additionally, we present the proportion of patients with a ≥ 50% reduction in MMD from baseline over the last 3 months amongst the subgroup of patients completing the DBTP on study medication (true completers).

### Statistical analysis

The FAS comprised all randomized patients who received at least one dose of the double-blind study medication.

In this post hoc sensitivity analysis, the efficacy of erenumab versus topiramate in patients who completed treatment with study medication was evaluated (true completers). However, this analysis would impair the randomization due to unequal discontinuation rates between the two treatment groups. To preserve the randomization, multiple imputation was conducted based on the data of patients who stayed on medication during the entire 24 weeks of the treatment phase. For those patients who discontinued the study medication, prospective data were imputed as if they had been able to stay on medication as well. Missing values from patients who discontinued the study were also imputed.

For the proportion of patients who achieved at least a 50% reduction in MMD, the odds ratio (OR) was calculated using a logistic regression model with categorical variables of treatment and stratification factor (MMD at baseline: 4–7 days, 8–14 days, ≥15 days).

The analysis model included the factors treatment, scheduled visit, and the stratification factor (MMD at baseline: 4–7 days, 8–14 days, ≥15 days). An unstructured covariance matrix for the random effects was assumed.

Statistical analyses were performed using SAS (version 9.2). Since both study drugs used are already on the market, no data monitoring committee was required.

### Ethics approval

At each trial center, the study protocol and its amendments were approved by an independent local ethics committee. Details of the study protocol and its amendments have been reported previously [27]. This study was conducted in compliance with Good Clinical Practice (GCP). Patients were informed according to GCP guidelines and provided written consent to participate in the trial.

The data were partly presented in abstract form at the International Headache Congress International Headache Society and European Headache Federation joint congress 2021 [29].

## Results

### Results from the HER-MES trial

Overall, of the 777 patients who were randomized (1:1) between February 2019 and July 2020, 389 patients were allocated to the erenumab group and 388 to the topiramate group. One patient in the erenumab group did not receive study medication and was therefore excluded from the analysis.

**Fig. 1:**
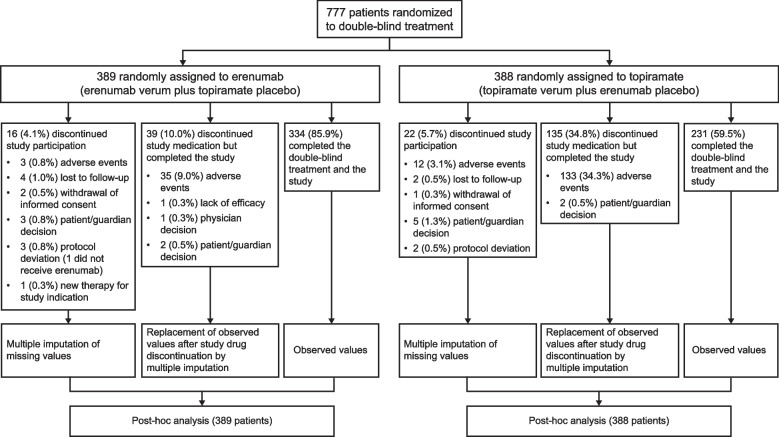
Treatment assignment, reasons for discontinuation and patients included in post hoc analysis

The treatment groups, reasons for treatment or study discontinuation and patients included in the post hoc analysis are presented in Fig. 1.

HER-MES demonstrated a favorable tolerability of erenumab over topiramate, as significantly less patients discontinued medication due to AEs in the erenumab group (10.6%) than in the topiramate group (38.9%) during the DBTP of the trial (OR: 0.19; 95% confidence interval [CI]: 0.13; 0.27; *p* < 0.001).

The primary analysis of the HER-MES results also showed that significantly more patients in the erenumab group achieved a ≥ 50% reduction in MMD from baseline (over the last 3 months of DBTP), demonstrating better efficacy of erenumab (55.4% vs 31.2%; OR: 2.76; 95% CI: 2.06; 3.71; *p* < 0.001) [27].

### Post hoc efficacy analysis

**Fig. 2:**
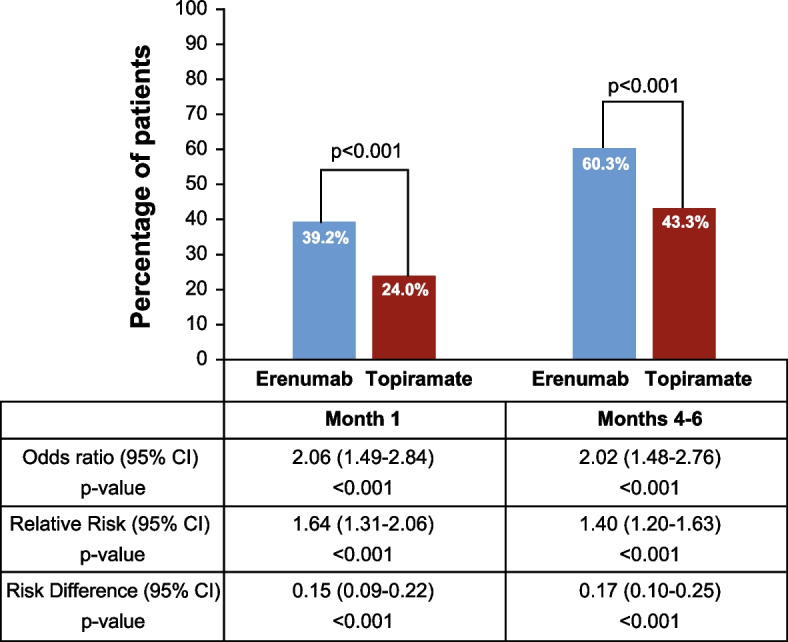
Proportion of patients who achieved at least a 50% reduction in MMD (post hoc analysis)

Patients on study medication until the end of the DBTP received a mean dose of 92 mg/day of topiramate (*n* = 231) and 119 mg/month of erenumab (*n* = 334). In the erenumab group, 60.3% (234/388) of patients achieved at least a 50% reduction in MMD during the last 3 months of the DBTP versus 43.3% (168/388) in the topiramate group (95% CI: 1.48; 2.76; *p* < 0.001). A significant difference was observed from month 1 of the DBTP with 39.2% (152/388) of patients in the erenumab group versus 24.0% (93/388) in the topiramate group (95% CI: 1.49; 2.84; *p* < 0.001) achieving at least a 50% reduction in MMD. Throughout the course of the DBTP, the odds of achieving at least a 50% reduction in MMD was about two times higher in the erenumab group compared to the topiramate group (Fig. 2).

The result of this post hoc efficacy analysis using the multiple imputation approach is similar to the 50% responder rate in true completers of the DBTP. In this subgroup of patients who stayed on treatment until study completion without multiple imputation (true completers), and thus breaking the randomization, 60.8% (203/334) of patients in the erenumab group and 45.9% (106/231) in the topiramate group achieved at least a 50% reduction in MMD during the last 3 months (months 4-6) of the DBTP (OR: 1.81; 95% CI: 1.29; 2.56).

### Effects of erenumab and topiramate on MMD

The mean number of MMD at baseline was 10.34 (4.05) days for erenumab and 10.46 (3.78) for the topiramate group. The mean change from baseline in MMD over the last 3 months (month 4, 5 and 6) of the DBTP was significantly higher in the erenumab group (− 6.13 days) compared to the topiramate group (− 4.90 days), with a mean difference of − 1.24 days (95% CI: − 1.87; − 0.61 days; *p* < 0.001; Table 1).

**Table 1:** Change from baseline in mean MMD over the last 3 months of the DBTP

	Erenumab (***N*** = 388)	Topiramate (***N*** = 388)	Mean Difference [95% CI]	***P***-value
N′	387	388		
Baseline Mean (SD)	10.34 (4.05)	10.46 (3.78)	not applicable	not applicable
Adjusted Mean Change (SE)	−6.13 (0.25)	−4.90 (0.27)	−1.24 [− 1.87; − 0.61]	< 0.001

*CI* Confidence interval, *N* Total number of patients, *N′* Number of patients in the analysis, *SD* Standard deviation, *SE* Standard error, *DBTP* Double-blind treatment phase, *MMD* Monthly migraine days

**Fig. 3:**
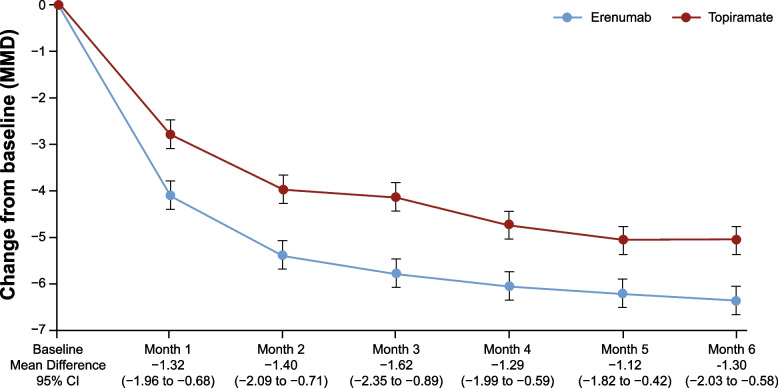
Change from baseline in MMD during the 24-week DBTP by month

During the first month of the DBTP, a significant difference in the MMD could be observed between erenumab (− 4.09 days) and topiramate (− 2.77 days: mean difference: − 1.32; 95% CI: − 1.96; − 0.68 days; *p* < 0.001). This analysis confirms the early onset of efficacy of erenumab as reported in several previous studies [30, 31] and the difference was maintained over the entire DBTP (Fig. 3).

## Discussion

The HER-MES post hoc analysis is the first direct head-to-head comparison in patients with migraine who completed 6 months of study treatment with either erenumab, a monoclonal antibody against the CGRP receptor, or topiramate, an oral medication for migraine prophylaxis. Erenumab led to a greater reduction in MMD from the first month onwards and demonstrated superiority in the proportion of patients who achieved a ≥ 50% reduction in MMD.

HER-MES was designed to assess the effectiveness of erenumab compared with topiramate [27]. Effectiveness is the combination of tolerability and efficacy, and it currently represents the best approximation of clinical reality under a controlled, randomized, double-blind study setting [32].

Most clinical trials focus on treatment efficacy as this is a requirement by regulatory authorities, imputing missing values due to tolerability issues via statistical methods like last observation carried forward (LOCF) or, more accurately, using multiple imputation. Consequently, for the HER-MES trial, describing for the first-time superior effectiveness of an anti-CGRP pathway inhibitor in a head-to-head trial approach, the question was raised as to whether the efficacy in patients who tolerated the study drug is also superior for erenumab. Therefore, we focused this analysis on the efficacy of erenumab versus topiramate in patients who completed 24 weeks on the study medication.

In this post hoc analysis of the HER-MES study, erenumab has shown superior efficacy over topiramate. Superiority was achieved from month 1 until the end of the DBTP, with an OR of 2.02 for the proportion of patients achieving a ≥ 50% reduction in MMD during the last 3 months from baseline. The early onset of superior efficacy from month 1 onwards can be explained by different aspects. Based on the s.c. mode of application the maximal plasma concentration of erenumab is much earlier reached as compared to topiramate, which needs to be up-titrated in order to reach a sufficient C_max_. In addition, monoclonal antibodies typically have a high affinity to their binding site which may also contribute to rapid onset of activity. Finally, due to the specificity of erenumab and very little unwanted effects, this monoclonal antibody can be administered in an efficacious dose from the start of therapy.

The average dose of topiramate in this post hoc analysis was 92 mg, which is rarely achieved in clinical practice due to tolerability issues.

Hence, presented data comprised patients who showed efficacy in a dose of 92 mg of topiramate, which is within the recommended range of topiramate for migraine prevention [16–18]. In addition, the results demonstrated that topiramate was an effective therapy for migraine patients tolerating the drug despite not as efficacious as erenumab.

In order to perform this analysis, only the values of patients on study medication were included. Data collected from patients after drug discontinuation were not incorporated. All missing efficacy values were replaced by multiple imputations based on efficacy results from patients on study drug to maintain randomization. Efficacy values were imputed for 40% of the patients on topiramate and 10% on erenumab. The result of this analysis using multiple imputation is similar to the 50% responder rate in true completers, while having the advantage of preserving the randomization of the study population.

Prior to this study, only a few direct comparisons of oral migraine prophylactics had been conducted [33]. The benefits of anti-CGRP pathway therapies were known from placebo-controlled trials [19–26]. The assumptions for the superior effects of anti-CGRP pathway monoclonal antibodies over oral migraine preventive therapies were derived from indirect comparisons only [34–36]. Here, we demonstrate the superior efficacy of an anti-CGRP pathway monoclonal antibody compared to an oral migraine prophylactic based on clinical head-to-head trial data for the first time.

## Conclusions

The HER-MES post hoc analysis confirmed the superior efficacy of erenumab over topiramate for patients on study medication, with a higher proportion of patients achieving a ≥ 50% reduction in MMD, reduction of MMD from baseline, and an early onset of action (significant superiority starting from month 1). The post hoc analysis of the HER-MES study further supports the initially published superior effectiveness results of erenumab compared with topiramate in the prevention of migraine across a broad patient population.

## Data Availability

The study data for the analysis described in this report may be made available on request by the author investigators or Novartis Pharma GmbH, sponsor of this clinical research.

## Abbreviations

AE: Adverse event
CGRP: Calcitonin gene-related peptide
CI: Confidence interval
CM: Chronic migraine
DBTP: Double-blind treatment phase
IHC: International Headache Congress
EHF: European Headache Federation
EM: Episodic migraine
FAS: Full analysis set
GCP: Good Clinical Practice
MMD: monthly migraine days
N: Total number of patients
N′: Number of patients in the analysis
OR: Odds ratio
RR: Relative risk
SD: Standard deviation
SE: Standard error
